# Coping strategies and perceptions of mental health services among women in South India

**DOI:** 10.1371/journal.pmen.0000142

**Published:** 2024-11-20

**Authors:** Lesley Jo Weaver, Alex Jagielski, Nagalambika Ningaiah, Purnima Madhivanan, Poornima Jaykrishna, Karl Krupp

**Affiliations:** 1 Department of Global Studies, University of Oregon, Eugene, Oregon, United States of America; 2 Public Health Research Institute of India, Mysuru, Karnataka, India; 3 Department of Health Promotion Sciences, Mel and Enid Zuckerman School of Public Health, University of Arizona, Tucson, Arizona, United States of America; 4 Department of Public Health Practice, Policy & Translational Research, Mel and Enid Zuckerman School of Public Health, University of Arizona, Tucson, Arizona, United States of America; University of Saskatchewan, CANADA

## Abstract

**Objectives:**

Lack of engagement with mental health services is a challenge for Global Mental Health research and intervention in lower- and middle-income settings. In India particularly, there is a significant treatment gap for people experiencing common mental disorders. This exploratory mixed-method study investigated women’s attitudes toward mental healthcare in Mysuru, India, and investigated what distress copings strategies they used in their everyday lives.

**Methods:**

We conducted qualitative interviews and administered a depression symptom screener with a community-based sample of 54 adult women. Interviews asked women to comment on their own distress experiences and stress management techniques, as well as their dispositions toward seeking mental healthcare for themselves or others.

**Results:**

Nearly 65 percent of the study sample screened for any level of depression risk, but only 5 had ever received mental healthcare. Around 20 percent of women stated that they would hypothetically be open to receiving such care. Yet, there was strong agreement across the study sample that mental healthcare was not an appropriate resource for addressing their own distress. Most women who rejected mental healthcare mentioned concerns about stigma and lack of perceived need or treatment inadequacy as their main reasons. Women described a broad range of coping strategies they used for dealing with distress, and which they reported as being effective. Many of these approaches resemble behavioral and talk therapies used in psychology and psychiatry.

**Conclusions:**

Women in this study generally did not view clinical mental healthcare as an appropriate treatment for their distress. Many already had effective strategies for managing their distress and analogous to existing psychological behavioral and talk therapies. A better understanding of why people reject mental healthcare is necessary for increasing the success of mental health interventions, and for developing new intervention approaches that support coping outside of clinical encounters.

## Introduction

Treatment uptake and adherence are persistent challenges for mental healthcare around the world [1]. Some of the best-supported, evidence-based randomized controlled trial studies for community-based depression and other common mental disorder treatment report that only 10% of eligible participants accept an offer of treatment [2]. Questions about how people in lower- and middle-income countries (LMICs) make decisions to engage (or not engage) with mental health services, and how they manage distress instead of seeking clinical treatment, have rarely been examined side-by-side. These questions are conceptually linked and are important, since for the last several decades, Global Mental Health practitioners have been building programs to address mental health treatment gaps in LMIC settings with mixed results [3].

Mental healthcare non-engagement in both HIC (high income country) and LMIC settings is often explained as a consequence of stigma, lack of perceived need, or lack of mental health literacy [4–16]. The WHO’s World Mental Health Surveys across 24 countries, for instance, concluded that “attitudinal barriers,” such as lack of perceived need and a desire to handle the problem on one’s own, were “much more important than structural barriers to both initiating and continuing treatment” for people with mild to moderate-level disorders [17]. As evidenced in the quote, this conceptualization of “attitudinal barriers” tends to place the onus of engagement (or lack thereof) on individuals living in LMICs, rather than interrogating how the system itself might make it difficult or irrelevant for people to engage with such treatment.

By contrast, research from critical global health and the medical social sciences argues that the problem of uptake is not a matter of “attitudinal barriers” that should be addressed at the individual level, but rather that the problem is inherent to the structures of mental healthcare delivery in LMICs, which often lacks appropriate cultural adaptation and may even unintentionally generate stigma by depicting the symptoms, significance, and nature of mental illnesses as grave and permanent: “once crazy, always crazy” [18, 19]. Public perception that mental healthcare is only for those with severe mental illness, which is especially common in LMIC settings (6), can generate stigma for anyone seeking treatment, regardless of the severity of their condition. This critical body of research argues that developing mental healthcare interventions with an emphasis on cultural relevance could improve uptake by increasing acceptability in cultures where mental healthcare seeking is otherwise stigmatized [20].

India has a robust set of mental health laws, including the National Mental Health Policy of 2014 and National Mental Healthcare Act of 2017, which affirmed the right to mental healthcare access, provided funds to improve that access, decriminalized suicide attempts, and affirmed the agency of people living with mental disorders to make their own healthcare decisions whenever possible. Yet the nation also has a very significant “treatment gap”—the difference between the number of people who need care and those who receive it—of up to 95 percent [21–24]. Even by generous estimates, India has only 0.75 psychiatrists available for every 100,000 citizens, and even fewer clinical psychologists [25]. In part for this reason, India has been a focal point for many Global Mental Health interventions designed to increase access to mental healthcare [26–29], including some of the early prototype studies that established a task-sharing model for addressing treatment gaps in LMICs [30]. That work laudably aims to increase access to clinical mental healthcare, but we do not actually know whether increasing access to care will translate to better health outcomes for people with common mental disorders in India because questions of cultural acceptability often get sidelined in the scramble to provide treatment where there is so much need.

Given India’s position as a nation with progressive mental healthcare legislation but limited access to care, and a strong tradition of research and intervention aimed at increasing access to care, a detailed understanding of why people choose to engage or not engage with clinical mental health services should be a priority [31]. In the several decades since the field of Global Mental Health was founded, it has made little population-level progress in closing the mental health gap [32–34], and a closer examination of what people do outside of clinical encounters may point to alternative approaches that could increase cultural sensitivity, uptake, and ultimately improve outcomes for people with mental illnesses.

The present study focuses on women specifically because we know that globally, gender is an important social determinant of mental health and illness [35], and the same is true in India [36]. Constraints such as lack of education, socioeconomic deprivation, intimate partner violence, personal or partner substance use, and dowry-related conflict predict common mental disorder symptoms among Indian women [37]. Women in India tend to emphasize social causes for distress and, likewise, engage in distress coping mechanisms that emphasize social reintegration and resumption of social roles that are valued in society, such as domestic caretaking [38, 39]. The limited existing evidence suggests that women and men alike in India rarely seek mental healthcare when experiencing common mental disorders, but may disclose symptoms of CMDs during healthcare appointments for other complaints [40, 41].

The present study aimed to investigate South Indian women’s attitudes toward mental health services and find out how they manage distress outside of clinical settings. It takes as its starting point a question posed long ago by Arthur Kleinman [42], when he coined the now classic idea of the category fallacy: *How might we support wellbeing for people who do not*, *as a whole*, *identify with psychiatric worldviews*? This question may have global implications, since in many parts of the world, psychiatric understandings of mental health and illness are not the dominant models people use to make sense of their distress [43, 44].

## Research design and methods

### Study setting and collaboration

This study took place in Mysuru (Mysore), India. Mysuru is a mid-sized city of about 1 million residents in the southwestern state of Karnataka and is home to the locally run partner organization for this project, the Public Health Research Institute of India (PHRII), which over the last 17 years has developed a strong reputation for delivering evidence-based interventions to reduce health disparities in the area. The first author co-conceptualized, designed, and oversaw field data collection in-person and remotely, while PHRII authors co-conceptualized, pilot tested, and performed data collection in collaboration with the first author.

### Study population and sampling

Women were eligible for participation if they were 18 years of age and older, Hindu, married or previously married, and spoke the regional language, Kannada. Women were purposively recruited through 17 of PHRII’s existing contacts in socioeconomically diverse neighborhoods, after which we employed snowball sampling to recruit additional participants one or two degrees of separation from the original contacts. This sampling strategy aimed to recruit a sample with caste and socioeconomic variation, but with relatively uniform life stage, religion, and language characteristics. Religious identity was confined to Hinduism because religion and language spoken at home are closely entwined in this context, and ensuring that study participants spoke a common language was important for other portions of the study. Because this study was designed to assess general community attitudes and perceptions around mental health, participants were not selected based on mental health status or prior experience with mental healthcare.

### Methods

From May 25, 2018 to June 21, 2018 and from August 25, 2022 to September 23, 2022, we recruited and conducted interviews in Kannada with a community-based sample totaling 54 women in Mysuru city. The initial series of interviews in 2018 were exploratory, asking women about the common causes of distress in their lives, eliciting Kannada terms and expressions for distress, and assessing where they would go for help if their distress felt unmanageable. The second phase in 2022, which was delayed because of COVID-19 disruptions, involved another round of individual interviews with a new sample of women. The research team repeated some questions from the 2018 work and added new ones exploring women’s coping, resilience, and knowledge and attitudes toward clinical mental healthcare. Knowledge, experience, and attitudes were assessed by a set of questions about how women would interpret symptoms of depression and anxiety presented in fictional vignettes, whether they would ever seek the help of a clinical practitioner if they had similar symptoms, whether they had ever sought such help, and if they knew of specific practitioners in the community. In both phases, interview questions were developed with the informal input of other researchers working in global mental health and in consultation with PHRII staff. The specific interview questions are published elsewhere [45].

Interviews occurred in women’s homes during the day when other family members were out, enabling women to speak with interviewers in private. Individual interviewing was chosen as the primary mechanism of data collection because in other study phases, women in group settings evinced some response bias and discomfort around disclosing personal experiences of distress in front of others. Interviews were conducted by a native-Kannada-speaking female member of the research team and typically lasted about one hour. Each participant was interviewed only one time.

At the conclusion of each interview, researchers administered a Kannada-translated and adapted version of the Patient Health Questionnaire-9 (PHQ-9) [46] and collected demographic data from each participant. The PHQ-9 asks participants to rate their frequency of nine depression symptoms in the previous two weeks using a Likert-type scale. A summary score was then calculated for each participant ranging from 0 to 27, indicating risk of depression. Clinical guidelines state that a score under 5 indicates no depression risk, 5–9 indicates mild risk, 10–14 moderate risk, 15–19 moderately severe risk, and 20–27 severe depression risk [47].

### Ethics statement

All study procedures were pre-approved by the first author’s US-based institutional review boards (University of Alabama #18-OR-177 and University of Oregon #00000606) as well as PHRII’s India-based institutional ethical review board (#2018-04-28-43 and #2022-08-13-75). Participants provided oral informed consent after the interviewer described in Kannada the purpose, procedures, risks, benefits, and confidentiality measures associated with the research. Participants received INR 300 to compensate them for their participation in interviews, plus coverage for travel costs in case they elected to travel to PHRII offices for the meeting. Those who screened positive for any depression risk were offered free and confidential mental health consultations with a psychiatrist or social worker, a gender-matched escort to appointments, free transport, and coverage of fees.

### Data analysis

Interviews were audio recorded and translated and transcribed from Kannada to English by a professional bilingual Kannada-English translator with a round of review and clarifications from the first author. Transcripts were then imported into Dedoose mixed-method analysis software [48]. Using an open coding approach with the constant comparative method, the first and second authors co-developed codebooks in an iterative fashion to reflect key themes in interview transcripts that addressed perceptions of clinical mental healthcare, distress experiences, and coping strategies [49]. These included core categories (e.g., "belief systems") as well as axial codes, which included sub-categories (e.g., "fate," “character grit”) and *in vivo* codes (e.g., "tension," an English term used to refer to generalized distress; [50]. The first and second authors cross-checked the other’s coding procedure for consistency.

Using Dedoose’s “descriptor x code” charts function, we analyzed relationships between stigma code frequency, PHQ score, and experience/willingness to engage with psychiatry. Dedoose’s normalize function allowed direct comparison between subgroups, which sometimes differed significantly in size (e.g., only 5 women reported having engaged with clinical care in the past). These mixed-method analyses allowed us to detect potentially meaningful differences in how frequently women discussed salient themes based on their mental healthcare usage and depression symptom severity.

Finally, we conducted basic frequency counts of how many women mentioned each of the key coping strategies we identified during the coding process. For some coping strategies, we were able to use a custom Python script to extract frequency counts. The script utilized the pandas library for data manipulation and analysis. A predefined list of search words or patterns, referred to as 'search_words,' was established. These search terms encompassed a range of coping strategies relevant to the study's objectives. For instance, the terms 'temple,' 'prayer,' 'pray,' 'pooja,' 'mantra,' 'recite,' 'mantras,' and 'God,' were used to identify religious coping strategies. The script then systematically searched for occurrences of the specified search words within the transcript and tallied the number of unique transcripts that mentioned the terms. For other coping strategies (e.g., cognitive reframing), it was not possible to identify keywords that would allow an exhaustive search using Python, so we performed hand-counts. These counts provided insights into the prevalence and distribution of coping strategies within the dataset.

## Results

A note on terminology: In this context, women did not differentiate between mental healthcare providers with psychology, psychiatry, MBBS (Bachelor of Medicine, Bachelor of Surgery, the terminal medical degree in India for physicians), or Master’s in Social Work (or similar) degrees held by counselors. They used English words like “psychiatrist” or “doctor” as catch-all terms to refer to any mental healthcare provider working in a clinical setting.

Table 1, below, displays demographic characteristics of the study sample and information on depression symptomology and knowledge and attitudes toward mental healthcare. Many women in this study experienced levels of distress that would be considered concerning; 64.8 percent of the total sample screened positive for any level of depression risk (PHQ score of 5 or above), while 29.6 percent screened positive for moderate or severe depression risk (score of 10 or above). Few population-based studies have estimated depression prevalence in South India, but the limited existing data suggest that a relatively high level of depression symptoms is common [51]. Women generally reported awareness of clinical mental healthcare resources in their community, with 45 (83 percent) affirming that they knew of its existence, and a subset of 26 (49 percent) concretely demonstrating that knowledge by being able to name a provider or share an experience of a first-degree contact receiving such care. Despite these relatively high levels of symptomology and awareness, a minority of women said they would be willing to engage with mental healthcare. Of the 54 women, 5 (9.3%) said they had previously received mental healthcare of one sort or another, while 15 (27.8%) said they would consider (or had considered) doing so if they found themselves in a situation where they felt their distress was unmanageable. There was overlap in these two subsamples. None of the 64.8 percent of women who screened positive for depression risk accepted the mental healthcare that was offered as part of the study. There was no significant variation in depression risk or attitudes toward mental healthcare between caste or socioeconomic groups in bivariate correlation and linear regression analyses, so these results are not reported. Nevertheless, in the results that follow, we characterize individuals by caste and depression symptom status to give readers a sense of the varied backgrounds of the study participants.

**Table 1:** Demographics, depression symptomology, and clinical mental health usage in the study sample (n = 54).

Indicator	Value	Range
Age (years, avg)	36.1	22–52
Hindu (%)	100	
Kannada speaking (%)	100	
Household members (avg)	3.9	2–11
Children living (avg)	1.6	0–4
Monthly household income (rupees, avg)	22,000	3,000–100,000
Employed outside home (%)	44.4	
Government caste designation (%)*		
ST (scheduled tribe)	3.7	
SC (scheduled caste)	9.3	
OBC (other backward caste)	22.2	
General	64.8	
PHQ-9 score (avg)	6.7	0–18
PHQ-9 score ‐ any level of depression risk (%)	64.8	
PHQ-9 score ‐ moderate or severe depression risk (%)	29.6	
Has received clinical mental healthcare (%)	9.3	
Would ever seek care (%)	27.8	
States knowledge of clinical mental healthcare (%)	83.3	
Demonstrates knowledge of clinical mental healthcare (%)	49.1	

*General castes are caste groups not eligible for government affirmative action programs; scheduled tribes, scheduled castes, and other backward castes are government-designated caste and ethnic groups eligible for affirmative action due to histories of oppression.

## Why women do not seek mental healthcare

Most women stated that they would not seek mental healthcare for distress, and their reasons fell into two main categories we will describe below: stigma; and perceptions that services are not relevant to their particular needs. After exploring these two themes in detail, we examine what coping strategies women use for distress.

### Stigma

Across the sample, 50 percent of women stated that mental healthcare seeking could be stigmatized; this included two women who had sought care themselves. When asked why it would be shameful to seek mental healthcare, women stated that there is a public perception that such care is only for people with severe mental illness, which they described as involving dissociative or psychotic symptoms and referred to using the term *hucca*, or “madness.” That perception spanned caste, income, depression risk, and employment categories across the study sample. As one lower-caste woman who screened negative for depression risk explained,

“People will think that they are mad [if they go to a mental healthcare provider]. That might be a reason [they would not go]. They might be worried that there might be problems in their life, that people will look at them as if they are mad, or mental. They might not go because of this.”

Similarly, a general caste woman who screened positive for moderate levels of depression risk said, “If I got to [mental healthcare] doctor, I feel like they may think that I am a madwoman. That is why they are not needed for me.”

As these quotes suggest, women’s concerns about stigma mainly centered on potential discrimination from others that could occur if people saw them seeking care, as opposed to self-stigma or stigma attached to the disease state [52]. A local pediatric psychologist confirmed this, saying, “In the small city of Mysore, the biggest challenge people report experiencing before seeking professional help is the taboo or stigma associated with it. This is called *huccha* (madness). They think seeking a psychologist will make people label them as *huccha*, and it negatively reflects on them in the community.” This provider’s comment also hints at the problem of associative stigma, or the stigma that attaches to people related socially or genetically to a person labeled *huccha*, which in this cultural context, can damage employment and marriage prospects [53, 54].

Mixed-method analysis suggested a pattern of more frequent discussion of stigma among the small number of women who reported having engaged with clinical mental healthcare (n = 5). A normalized code x descriptor analysis, displayed in Table 2, demonstrates that those who had previously engaged with mental healthcare had higher relative frequencies on the stigma code than either those who would consider engaging (but had never done so), or those who would not consider engaging. There were 60 mentions of stigma across the 5 interviews with people who had engaged mental healthcare, in contrast to only 49 mentions of stigma across the 33 interviews with people who had not engaged mental healthcare. Keeping in mind that these results are limited by small sample sizes, it could be the case that women with direct experience of mental healthcare also have more direct experience of the stigma associated with that healthcare, or that women who had sought mental healthcare had greater stigma experience because their symptoms were more severe.

**Table 2:** Mixed-method analysis comparing frequency of stigma mentions with participant’s experience with mental healthcare. Results produced using a normalized code x descriptor analysis in Dedoose mixed-method analysis software.

Has engaged mental healthcare	Frequency of mentioning stigma during interviews (summed across each category)
No (n = 33)	49
Yes (n = 5)	60
Unstated (n = 1)	0
No, but would consider seeking it (n = 15)	32

Instead of going to mental health clinics, women often expressed a preference for seeking help from *mahila sanghas*, or women’s empowerment organizations. One general caste woman with mild depression symptoms said, “These *mahila sanghas* should help me secretly because my respect in the society should not go, and all my family issues and problems should not be disclosed to everyone. They can help me by suggesting how others can be punished or how I should lead my life. … *Mahila sanghas* will give solutions by maintain the confidentiality.” Another general caste woman with mild depression risk reported that if she were experiencing depression or anxiety, “I would go to *mahila sanghas*. One of my in-laws works there. They will actually guide people in problems to have a way out. They will tell people individually like where to go, or what to do, or what to study.” Likewise, a third woman with no depression risk said that if someone were suffering, they could go to such an organization.

“She must tell everything without hiding anything. She must tell every scene what has happened with her. There are many *mahila sanghas* and NGOs to support. In olden days, if there were any problems, it was necessary to solve by ourselves. Otherwise, one must commit suicide; that was the mentality. Now it is not like that. People are educated; they can share it with family members or neighbors. Or else they can go to *mahila sanghas*. Through them they can approach the police and solve the problem.”

### Perceptions that services are not relevant to their needs

Besides concerns about stigma related to care-seeking, women said they would decline mental healthcare because they felt they could handle their problems on their own. Women differentiated between the kind of person who might need mental healthcare and the kind who would not, emphasizing that some people are inherently “weak-minded” while others are resilient and strong. Those who were “weak-minded” were prone to descend into madness when over-stressed and would require outside help to manage their distress. “People who are mentally weak will usually go to counselor,” explained one general caste woman with no depression risk. “If they do not go [on their own], their family will take them. …Some people cannot find answers on their own, but when somebody else tells them the reason for their problem, they will realize quickly.” Women in this study almost universally characterized themselves as “strong-minded,” and they often relied on this as a reason for why they would not want to seek mental healthcare. Women positively characterized “strong-minded” people as careful and deep thinkers, acting with self-conviction or self-efficacy to “manage” things without help. This personal resilience was perceived as a valuable quality; several women articulated the idea that adversity begets strength. One woman, who had one of the highest depression risk scores in the study, used an idiom to explain that idea.

“My mother has taught me that if we suffer now, then in the future we can grow strong. The stone that suffers when it is struck by an artist will someday become a lovely sculpture. So, I think I can handle all this [depression or anxiety symptoms]. I have handled many big things in my life, and so I could handle this also.”

Even when women’s distress would be considered relatively severe by psychiatric standards, they described relying on their qualities of “strong-mindedness” to manage distress on their own. One woman who had considered suicide in the past and screened positive for moderate depression risk explained, “I felt like committing suicide; I cried and cried a lot. Then thought of my two kids. Who will look after them if I take any bad decision in a hurry? I consoled myself, took courage on my own.” Through this process of remembering her responsibilities and attachments to her children, she was able to turn her mind away from suicide without outside help. She perceived this as a positive approach for handling her symptoms.

Other women felt they did not need mental healthcare because they viewed their problems as resulting from external stressors that could not be addressed by clinical intervention. “When there are any stressors at home, or if there are any problems, it is from money,” one said. Another explained, “[A main] reason for stress is money. Not only for me; money is the thing which causes stress to every human being. It may be [a major] problem for some, and small for others. But money is the reason for stress.” Women were acutely aware that mental health clinicians could not solve their money problems.

There were no discernable patterns between women’s willingness to engage with clinical care and their depression symptom scores in mixed-method analysis. In other words, women’s general lack of interest in clinical care did not appear to be patterned by their own levels or personal experiences of distress. It might have been the case that women would seek mental healthcare under different conditions than those we assessed in the study, but they generally agreed that the kinds of distress we were talking about in this study—specifically, symptoms of clinical depression and anxiety—were not an appropriate reason to seek it.

### Coping strategies

Since most women did not desire mental healthcare, we explored what strategies they used to manage their distress instead. Women’s coping strategies were diverse, and we were able to group them into 5 major categories: cognitive reframing, somatic release, self-talk, mindfulness activities, and social engagement (see Table 3, below). There was some overlap between categories; for example, visits to temples could serve both a religious coping and a social engagement function; breathwork could be considered mindfulness or somatic release. Below we describe the nature and frequency of each category with exemplar quotes.

**Table 3:** Categories of coping strategies used by women in the study, and frequency with which they were mentioned.

Category	Description	Frequency
**Cognitive reframing**	Solution-focused and positive thinking	44 percent (n = 24)
**Somatic release**	Crying, laughing, or doing hard physical work	76 percent (n = 41)
**Self-talk**	Speaking to one’s own mind, speaking to an imaginary person, or writing down one’s thoughts and feelings	13 percent (n = 7)
**Mindfulness and religious activities**	Meditation, yoga, and breathing exercises; praying, visiting temples, or doing *pooja* (worship)	22 percent (n = 12; mindfulness); 65 percent (n = 35; religious activities)
**Social engagement**	Sharing problems with close friends or family members	91 percent (n = 49)

#### Cognitive reframing

Forty-four percent of women across the study sample practiced a type of cognitive reframing where they intentionally steered their minds toward solution-focused and positive thought patterns instead of ruminating. “First, I must have patience,” explained one general-caste woman who screened negative for depression risk. “I will think of whether I can solve this on my own or not. If there is a problem, it has a solution. So I will think over the solution on my own.” Deliberately changing one’s thinking was a common theme. A scheduled caste woman with no depression risk said, “I feel It will become alright [when I experience a stressful incident]. I change my own mind. I sometimes feel that, why should I be worrying about loans because I have six months [to repay them], and I can always mortgage my jewelry. …I can reduce my tension like this.” Similarly, a general-caste woman who screened positive for moderate depression risk explained, “I manage by thinking about [my problems]. It has to be me who can set things right; no one else will come to repair that. Others can only give suggestions.”

Women who endorsed cognitive reframing often thought of themselves as brave or courageous, even sometimes using the construct of having a “strong mind” to characterize themselves. “I have courage, madam” said one general caste participant with no depression risk. “I will rest over there, if I want to forget [my troubles].” When asked what advice she would give to women like herself dealing with depression or anxiety symptoms, she simply replied, “I would tell them to be positive, and tell them not to take all of this to heart.” Another general caste woman with moderate depression risk explained, “I have to get out of the stress and difficulties [on my own]. We have to bring ourselves on the right path to live among the society and family.” The positive moral undertones here were evident.

#### Somatic release

Seventy-six percent women said that they relied on some form of somatic release, such as crying, laughing, or doing hard physical work, to manage distress. They explained that physical release forced a change of state, allowing them to shift toward clearer thinking about their problems and solutions. One lower-caste woman with moderate depression risk stated, “I developed this strategy on my own: I laugh when I am angry. …Yes, I just laugh. [I do it] to calm myself down.” Another general-caste woman with low depression risk said,

“When I face challenges, I first think about how I can approach it. When it is not possible for me, I will sit by myself and cry loudly; I will sit by myself and cry loudly that this thing happened. After that, I think, if I just sit here because of what has happened, who will look after my life? I should do things in my own life. I should eat my own food; it is not possible to go and have other people’s food all the time. I have to live for that. I have my own responsibilities.”

Among the various somatic release strategies that women used, crying was by far the most common. When she felt distressed, explained a general caste woman who screened negative for depression risk, “I start trembling and crying. Then after I cry a lot and cool down, my mind starts working. Until then, it does not work. I will then start thinking that I have to do like this, and not the way I have done it [previously].” Women reported feeling relief after crying and being able to approach their problems with greater clarity.

#### Self-talk and journaling

Thirteen percent of women reported that they spoke out loud to themselves or an imaginary person, or wrote down their thoughts and feelings, to cope with distress. “I will talk with myself,” said one lower-caste women who screened for high depression risk. “Yes, I talk with myself. I imagine someone who is close to me, and then talk to them; like this I share all my emotions. …When I tell everything to that imaginary person, then I am relieved.” A general caste woman with no depression risk explained, “When I am alone and do not have my mind in control, I will talk to myself. I don’t talk out loud; I talk to myself in my mind.” Another general caste woman who screened for moderate depression risk explained, “Sometimes I will write all my feelings down on a paper. My teacher had taught me to write our feelings on a paper. …Even six months ago I wrote a letter like that in the name of God. Then I just threw it away.” For this smaller group of women, verbal or written self-expression provided an outlet that did not rely on the presence of another person or their willingness to hear her complaints.

#### Mindfulness and religious activities

We categorized mindfulness and religious activities together since practices like meditation and breathwork, which have been adopted in Western secular contexts as “mindfulness activities,” are often not conceptualized as separate from religious activity in South Asian religious traditions. Twenty-two percent of the study sample said they used meditation and yogic breathing exercises to manage their distress. They described activities like “doing meditation or sitting calmly for half an hour,” or, in one case, listening to recordings of calming piano music. One general caste woman with low depression risk described yogic breathing as a coping strategy. “One way to inhale and another way to exhale—that’s *pranayama* in yoga—that type of breathing reduces my stress and helps me feel relaxed.” Sixty-five percent of women described praying, visiting temples, or engaging in *pooja* (worship), which typically involves chanting mantras and making offerings to deities in a meditative state. One general caste participant with low depression risk explained, “I change my thought process [by going] to temple, offering prayers, doing *pooja* at home, sitting and crying in front of God himself.”

#### Social engagement

Finally, women relied on social support to manage distress. The vast majority—over 90 percent—described sharing problems with close friends or family members as a first-line strategy for coping, venting emotions, and problem-solving. “Most of the time, I share my feelings with my husband,” said one lower caste woman who screened negative for depression risk. “If I sit in front of him and talk, I start crying, so we usually message each other [instead]. When he goes to his work and he is on WhatsApp, I share my feelings.” While this woman’s husband was a source of support, many others did not get along so well with their husbands and instead turned to female friends. “To get peace of mind, I sit with four or five people and start to make some jokes. The reason is, I have to solve my problems on my own. …So I start to share jokes and I also laugh with them and get peace of mind,” said a general caste woman with moderate depression risk. Although this participant did not feel that her friends could solve her problems, she took comfort in their humor to lift her mood. She also felt that service to others helped her feel better when distressed. “I go and help some people who are in trouble. When I help someone, I get peace of mind. Whatever problems we may have, we should still always help others,” she explained. Another woman commented, “With my friends, that is where I find happiness. Laughing with friends and joking with them, crying with friends. Everything from happiness to sadness [I can share] with my friends.” A general caste woman with no depression risk explained the importance of sharing problems idiomatically. “Sometimes, they say, sorrows will become a rock if they are kept in the mind,” by which she meant that troubles will linger and even solidify into permanent states if not shared with others. Many women described getting out of the house to visit family members or friends as a helpful strategy to shift their mindsets away from troubles.

## Discussion

One of the framing questions of this study was, Why might women reject or avoid clinical mental healthcare?

We found two main reasons why women either rejected or avoided seeking clinical mental healthcare. The first reason was women’s concerns about stigma, but not in the usual sense of stigma toward people with mental illness. The second reason was women’s perception that mental health services are not relevant to their needs. Below, we discuss these concerns in turn.

### Stigma toward clinical encounters, not toward distress

Social stigma toward mental disorders is often cited as a key reason for lack of mental healthcare seeking in research conducted in LMIC settings [4–14, 16]. Stigma was indeed an important driver of women’s rejection of mental healthcare in this study. But unlike most of the extant literature, which describes stigma that attaches to individuals for being mentally ill [15, 55], women in this study associated stigma with the act of seeking clinical care, not with states of distress. This is similar to the findings of Koschorke and colleagues, who report that service users across seven LMIC settings experienced discrimination from family and community directly related to their care-seeking behaviors (for instance, one woman in Tunisia reported, “Even my best friend, said ‘you’re crazy, you go to [name of psychiatric hospital], your siblings are crazy, your whole family is crazy. Who would marry into your family? Who would marry you? If you’re a crazy person who seeks treatment at [name if psychiatric hospital], who would marry you?” [6]. Our mixed-method analyses revealed a similar pattern, where women in our study who had attended clinical mental healthcare in the past mentioned stigma far more frequently than those who had not sought care; the association between clinical care and stigma was also a common theme in the qualitative analyses.

Women did report that stigma attaches to individuals with severe symptoms associated with psychosis (e.g., hallucinations, delusions), but they did not associate this stigma with individuals experiencing symptoms of common mental disorders (CMDs) like depression and anxiety. Stigma arose primarily because of the perception that clinical mental healthcare is for those who are “mad,” which in this context usually referred to people with dissociative and/or psychotic symptoms. While other scholars have noted a similar pattern of stigma around care-seeking [6], to our knowledge, this is one of the first studies to find that the stigma of care-seeking eclipses the stigma of CMD-type distress.

Interventions to increase access to mental healthcare often involve a psychoeducation component designed to convey the idea that common mental disorders are disease states for which effective clinical treatments exist [56, 57]. The intent of these programs is usually to destigmatize mental illness and increase mental health literacy, but by connecting people with distress to care systems that are stigmatized, psychoeducation could paradoxically increase stigma in this cultural context. That phenomenon has been observed in some Western settings where disease-model psychoeducation has reproduced stigma by reifying mental illness as a static state [18, 58].

These findings demonstrate that simplistic assumptions about the way stigma operates should not overshadow careful study of where stigma comes from and what it attaches to. For our study participants, distress itself was not the problem; the stigmatized clinical setting was the problem.

Given the stigma that attaches to clinical mental healthcare in this setting, study participants suggested alternatives where women could seek support without stigma. They strongly endorsed women’s empowerment organizations, or *mahila sanghas*, as a positive alternative to clinical settings. They described these organizations as welcoming, confidential, and trustworthy, and often recommended that if a woman was experiencing more distress than she could handle, she should make a visit to a *mahila sangha* as a first or second step for seeking help.

### Perceptions that services are not relevant to their needs

In biomedical psychiatry, symptoms are generally considered severe if they interfere with everyday functioning (e.g., a woman is so depressed that she can’t get out of bed in the morning). But women in this study conceptualized “severe” not in terms of disability, but in terms of “madness.” They distinguished between people who were “weak-minded” and “strong-minded”; weak-minded people were prone to mental illness and even madness, while strong-minded people were self-reliant and generally viewed positively. Women normalized their distress through belief systems that included viewing suffering as an inherent part of life and valuing suffering as a source of personal growth and strength. Given that women almost always described themselves as “strong-minded,” they generally did not consider themselves candidates for mental health treatment, even when their distress was severe. Our finding echoes the results of previous studies addressing attitudes toward clinical mental healthcare in South Asian populations, which report lack of perceived need as a key driver of mental healthcare non-engagement [31, 59]. Moreover, several previous studies report lack of perceived need as a key deterrent to mental healthcare seeking in other LMIC settings, suggesting that this is not an India-specific phenomenon [4–7, 9, 10].

In some cases, women did not see themselves as candidates for mental health treatment because their distress arose primarily from social conditions such as poverty, and they knew a clinician could not amend these causes. This is similar to the findings of some previous work in India, which argues that mental healthcare may lack relevance in contexts where people’s distress arises from basic needs insecurity [31].

A second guiding question of this research was, What do women do to manage distress instead of engaging clinical mental health services?

We found that women described a diverse array of dispositions and techniques to manage distress. These consisted exclusively of self-directed activities they engaged at home or in their communities, not in clinical spaces, likely a result of the stigma associated with care-seeking that we described above. Since this study was cross-sectional, we could not determine whether women’s coping activities actually resulted in better mental health outcomes, but they reported that they found these strategies effective for managing distress.

The two most common coping strategies in this study were social engagement and mindfulness/religious activity. These results align with a large body of work in the medical social sciences demonstrating the importance of social integration for mental health [60] and the value of religious and spiritual resources for coping in resource-constrained settings [61]. These findings also align with the long history of research documenting the role of faith healers who may function in positive ways as front-line healthcare workers for both physical and mental health needs across India, and as a bridge between patients and medical providers when needed [62–64].

During data analysis, the research team noticed that many of the coping strategies women described resemble current evidence-based movements in psychology and talk therapy; this was a post-hoc observation rather than a guiding principle for the identification of coping themes. For instance, women’s discussion of somatic release mirrors recent trends in psychotherapy such as Somatic Experiencing [65] and Tension and Trauma Releasing Exercises [66], trauma therapies that differ from cognitive approaches by encouraging clients to focus on bodily sensations and physical release of tension. These relatively new, “bottom-up” or “body-first” approaches to emotional processing like Somatic Experiencing have positive impacts on symptoms and perceived wellbeing among traumatized and non-traumatized individuals [67].

Likewise, women’s strategy of self-talk and solution-focused thinking mirrors acceptance and commitment therapy (ACT), a form of pragmatic therapy that emphasizes changing one’s self-talk to support positive actions rather than the more traditional cognitive approach that involves changing thought patterns [68]. ACT appears to be as effective for treating depression and anxiety as cognitive therapy, but it functions through different mechanisms [69].

Women’s discussions of changing their mindsets are reminiscent of cognitive reframing, a key skill taught in cognitive-behavioral therapy which encourages people to shift their mindsets to see a situation in a different perspective [70].

Finally, women’s endorsement of social interaction as an effective coping strategy echoes the decades of research demonstrating that social connectedness buffers mental health while social isolation damages it [60]. An approach to stress management focused on social engagement also mirrors the research on interpersonal emotional regulation, which is a key mechanism of social support for people with depression and could be harnessed therapeutically in group sessions [71–73].

We point out these similarities between women’s coping strategies and existing therapies not to imply that women have no need for mental health support, but rather to challenge the common assumption that people’s autochthonous coping strategies are distant from (and therefore perhaps less effective than) clinical approaches. The similarity between women’s copings strategies and therapy best practices implies that women in this cultural context might be receptive to teaching and learning these kinds of therapies in more structured modalities, as long as they did not rely on clinical settings. These results could provide a road map for developing culturally acceptable interventions that incorporate preexisting ways that people manage their distress outside of clinical encounters.

### Reimagining interventions that build on women’s existing strategies

The diverse coping strategies women identified in this study support a conclusion that cultural psychologists have long made: that people’s coping strategies should be taken seriously as sources of accessible, low-stigma interventions [43]. Not everyone would necessarily have mastery of all the coping strategies identified in this study, but these could be taught and learned in culturally-acceptable settings by leaders such as community health workers or nurses, who could be physically located outside clinical settings and might work with groups of individuals in a manner similar to task-sharing interventions in India [30]. Given that many of women’s existing strategies resemble existing therapy practices, interventions could use culturally modified versions of existing training manuals or could develop culturally derived materials for use in specific cultural contexts. An approach like this could stand alone, or it could be paired with additional innovative treatment modalities to comprise a wellbeing-focused psychosocial intervention [56]. Given women’s apparent disinterest in interventions focused narrowly on clinical symptoms in this study, more holistic interventions focused on wellbeing and social integration might meet greater success.

Based on our results, any intervention would need to seriously consider working outside clinical settings, possibly in a women’s empowerment organization or *mahila sangha*, which women were familiar with and endorsed as trustworthy and comfortable settings. Clinical settings provide obvious logistical advantages for researchers, and current best practices even suggest that interventions in LMIC settings should build on existing health infrastructure [74]. Women’s discomfort with clinics in this context, however, suggested that the benefits of convenience and cost-savings from working in clinics might involve serious tradeoffs in terms of women’s willingness to engage. Our results suggest that research and interventions that take place outside clinic settings would have significantly higher uptake than those confined to clinical settings.

### Limitations

Our results should be interpreted in the context of the fact that no study participants were suffering from acute mental health crises, even though many did report symptoms of depression. Because of the sampling strategy, Christian, Muslim, and other religious minority women were not represented, nor were youth. Therefore, the results are not generalizable to Indian women in general or to people under the age of 18, who in this context may be especially vulnerable to common mental disorders [75].

## Conclusions

This study explored South Indian women’s perceptions of mental health services and their coping strategies for everyday distress. Although women in the study experienced distress and were aware of mental healthcare resources available in their community, many rejected those supports. This rejection was partially related to their perceptions that clinical mental healthcare was stigmatizing in this cultural context, but also partially related to the fact that they did not perceive themselves as needing such treatment. Often, women felt that they were not in need of external help or that the social or structural origins of their distress could not be addressed in a clinical setting. Instead, women relied on coping strategies that they endorsed as highly effective, many of which resemble contemporary psychotherapy approaches. These findings raise questions about how the field of Global Mental Health could develop research and interventions that support wellbeing for people who do not identify or interpret their own experiences through psychiatry. We argue, along with other scholars [56], that Global Mental Health research and intervention agendas could be better informed by how people cope outside of clinical settings. This would be true for both LMIC settings like the one in which this study took place, and also for higher-income settings. The present study provided clear examples of how people cope without clinical mental healthcare, and how and why they might reject clinical interventions in favor of autochthonous coping strategies.

## Supporting information

S1 Checklist Inclusivity in global research. (DOCX)
